# Supplementary material for: A genome-wide association study finds genetic variants associated with neck or shoulder pain in UK Biobank
Source: Hum Mol Genet. 2020 Apr 3;29(8):1396–404. doi: 10.1093/hmg/ddaa058 (PMC7254846; doi:10.1093/hmg/ddaa058)
Supplement: Supplementary_Figure_S1_ddaa058 [file supplementary_figure_s1_ddaa058.pptx]

## Slide 1
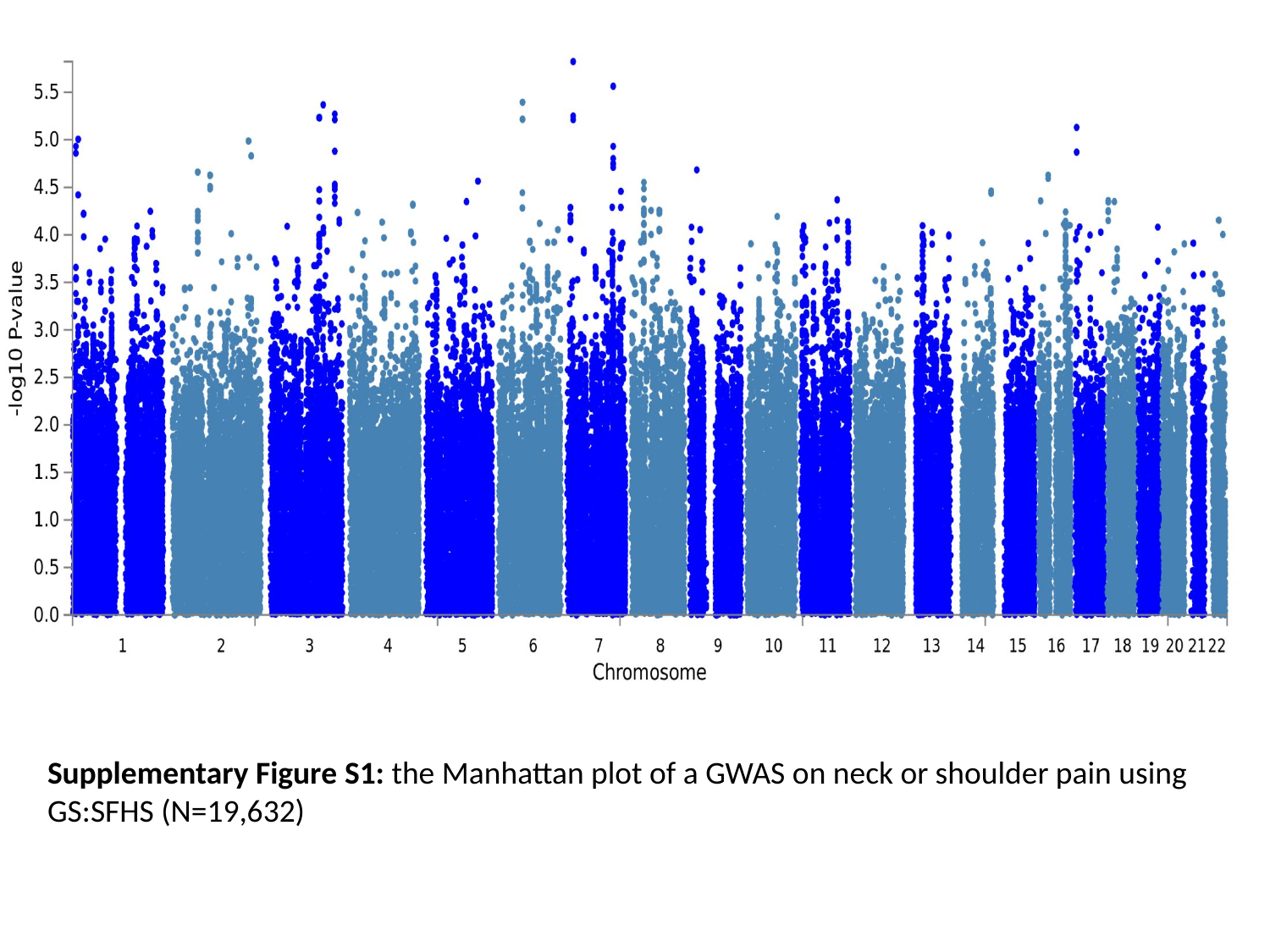

Supplementary Figure S1: the Manhattan plot of a GWAS on neck or shoulder pain using GS:SFHS (N=19,632)
