# Supplementary material for: A genome-wide association study finds genetic variants associated with neck or shoulder pain in UK Biobank
Source: Hum Mol Genet. 2020 Apr 3;29(8):1396–404. doi: 10.1093/hmg/ddaa058 (PMC7254846; doi:10.1093/hmg/ddaa058)
Supplement: Supplementary_Table_S1_ddaa058 [file supplementary_table_s1_ddaa058.docx]

**Supplementary Table S1.** Clinical characteristics of neck or shoulder pain cases and controls in the GS:SFHS and TwinsUK

|  | GS:SHFS |  | TwinsUK |  |
| --- | --- | --- | --- | --- |
| Covariates | Cases | Controls | Cases | Controls |
| Sex (male:female) | 2131 (66.9%) : 1056 (33.1%) | 9444 (57.5%) : 6967 (42.5%) | 133 (7.2%) : 1715 (92.8%) | 191 (9.0%) : 1943 (91.0%) |
| Age (years) | 52.6 (12.9) | 46.4 (15.3) | 52.2 (12.4) | 51.7 (13.2) |
| BMI (kg/m^2^) | 27.5 (5.5) | 26.6 (5.2) | 25.8 (4.8) | 26.3 (3.6) |

BMI: body mass index

A chi-square test was used to test the difference of gender frequency between cases and controls and an independent t test was used for other covariates.

Continuous covariates were presented as mean (standard deviation).
